# Combinatorial metabolic engineering of *Mycobacterium neoaurum* LY-2 for efficient androstenedione production from phytosterols

**DOI:** 10.1016/j.synbio.2026.08.003

**Published:** 2026-08-19

**Authors:** Liyang Xu, Chuxian Peng, Wenjing Pan, Yingjia Tong, Jinsong Shi, Zhenghong Xu, Hui Li

**Affiliations:** a School of Life Sciences and Health Engineering, Jiangnan University, Wuxi, 214122, China; b College of Biomass Science and Engineering, Sichuan University, Chengdu, 610065, China

**Keywords:** *Mycobacterium neoaurum*, Androstenedione, Phytosterols, Metabolic engineering

## Abstract

Androstenedione (AD) is an important precursor for the synthesis of corticosteroids, sex hormones, and other high-value steroid pharmaceuticals. Microbial conversion of phytosterols provides a sustainable route for AD production, but current production levels remain insufficient for practical industrial application. In this study, a modular metabolic engineering strategy was developed to improve AD biosynthesis from phytosterols in *Mycobacterium neoaurum* LY-2. Competing C19 and C22 steroid pathways were first blocked to attenuate AD degradation, generating the AD-accumulating strain Mbn12 with a titer of 5.14 g/L. Sterol catabolic flux was then enhanced by relieving transcriptional repression and improving acetyl-CoA and propionyl-CoA utilization, which increased AD production to 6.08 g/L. To support high-flux sterol oxidation, cofactor regeneration and oxidative stress resistance were further strengthened, raising AD production to 6.67 g/L. Finally, moderate cell envelope remodeling improved substrate transport and increased AD production to 6.98 g/L. The final engineered strain Mbn30 produced 20.55 g/L AD from 30 g/L phytosterols in a 5 L fermenter, representing a 48.6% improvement over Mbn12. This work provides a scalable engineering framework for AD production and offers guidance for the microbial manufacture of other steroid intermediates.

## Highlights

• C19 and C22 pathways were rerouted for selective androstenedione accumulation.
• Transcriptional control and acyl-CoA balance enhanced phytosterol flux.
• Redox regeneration and envelope remodeling increased AD production to 6.98 g/L.
• The highest AD titer reached 20.55 g/L in a 5 L fermenter.

## Graphical abstract

**Figure:**
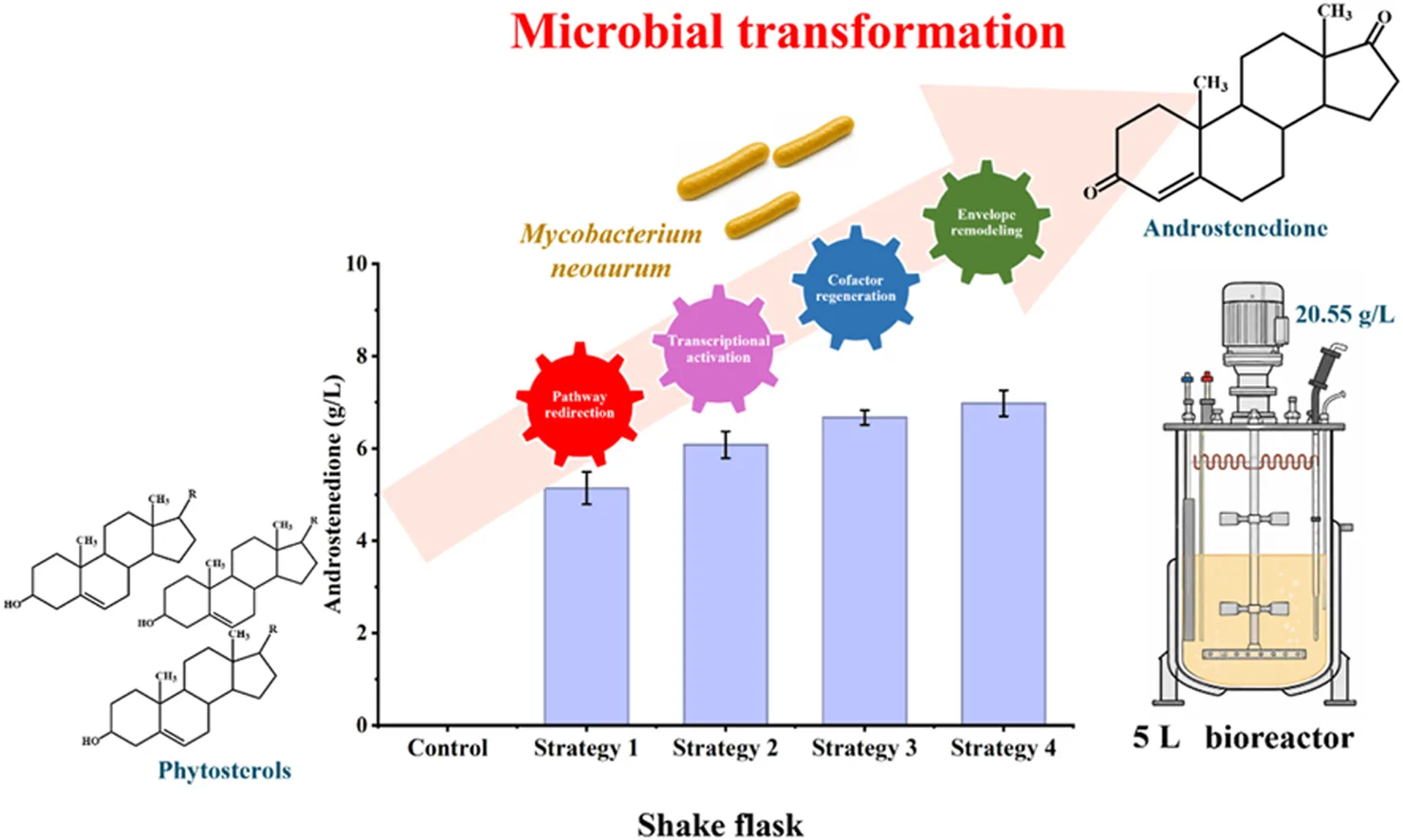

## 1 Introduction

Steroidal drugs represent the second largest class of pharmaceuticals after antibiotics and play important roles in anti-inflammatory, anti-allergic, contraceptive, hormone replacement, and anticancer therapies [1,2]. Androstenedione (AD) is a key C19 steroid intermediate for the synthesis of multiple steroid hormones, and its demand has continued to increase with the expansion of steroid drug manufacturing [3,4]. Compared with traditional routes based on natural sapogenins such as diosgenin, microbial transformation of phytosterols provides a more sustainable alternative because phytosterols are abundant, inexpensive, and renewable feedstocks [5]. Among steroid-transforming microorganisms, *Mycobacterium neoaurum* is particularly attractive because it naturally possesses sterol uptake, nucleus oxidation, and side-chain degradation systems, enabling the production of AD, androst-1,4-diene-3,17-dione (ADD), 9α-hydroxy-4-androstene-3,17-dione (9α-OH-AD), and 22-hydroxy-23,24-bisnorchol-4-ene-3-one (HBC)-type intermediates from phytosterols [[6], [7], [8], [9], [10]]. However, phytosterol degradation in *M. neoaurum* involves complex C19 and C22 metabolic branches, in which AD can be further converted into ADD, 9α-OH-AD, or C22 byproducts, thereby limiting its accumulation to levels required for large-scale production.

Previous studies on AD production from phytosterols indicate that efficient AD accumulation requires coordinated regulation of the overall sterol catabolic flux. Early efforts mainly improved AD production through fermentation optimization and substrate solubilization. For example, Zhang et al. produced 5.96 g/L AD from 8.98 g/L phytosterols using *M. neoaurum* ZJUVN-08, but the process strongly depended on hydroxypropyl-β-cyclodextrin-assisted substrate utilization [7]. As sterol degradation pathways have been progressively elucidated, metabolic flux rerouting has become a key strategy for directing phytosterol-derived intermediates toward target steroid products. Engineering steroid degradation pathways has been shown to improve 9α-OH-AD accumulation, while balancing side-chain degradation with 9-position hydroxylation further enhances product purity and yield [8,11]. For C19 and C22 branch regulation, deletion of 3-oxo-4-pregnene-20-carboxyl-CoA reductase (*opccR*) and steroid aldolase (*salA*) reduced C22 by-product formation and improved ADD molar yield, and functional analysis of SalA confirmed its role in C24 side-chain cleavage and 4-HBC formation [12,13]. In parallel, strengthening sterol side-chain degradation and central metabolic capacity has also been used to increase precursor supply for AD biosynthesis. For AD production, Deletion of fatty acid degradation regulator (*fdmR*) combined with central metabolic reinforcement enabled 9.29 g/L AD production within 72 h, while enhancement of propionyl-CoA metabolism further improved AD conversion efficiency [14,15]. These studies suggest that high-level AD production from phytosterols requires global coordination of sterol catabolic flux rather than independent optimization of a single pathway module.

In addition to metabolic flux distribution, efficient phytosterol biotransformation is constrained by several non-pathway factors, particularly substrate bioavailability, intracellular cofactor regeneration, and cell envelope permeability. Phytosterols are highly hydrophobic substrates, and their poor dispersion in aqueous fermentation systems often limits their contact with mycobacterial cells and reduces subsequent uptake and conversion efficiency [8,11,16,17]. To overcome this limitation, Shen et al. investigated the effect of hydroxypropyl-β-cyclodextrin on phytosterol biotransformation by different *M. neoaurum* strains and showed that cyclodextrin enhanced sterol conversion by improving substrate dispersion and bioavailability [16]. Gao et al. further combined substrate pretreatment with metabolic engineering to improve phytosterol accessibility, thereby increasing AD production efficiency [14]. However, these strategies mainly alleviate extracellular mass-transfer limitations and cannot fully resolve intracellular catalytic pressure or transmembrane transport barriers. Within the cells, sterol side-chain degradation involves multiple FAD- and NAD^+^-dependent oxidation reactions. Enhanced catabolic flux increases the formation of FADH_2_ and NADH, and insufficient reoxidation of these reduced cofactors may disturb intracellular redox balance and promote reactive oxygen species or H_2_O_2_ accumulation [[18], [19], [20]]. Song et al. increased intracellular FAD supply by overexpressing 3,4-dihydroxy-2-butanone 4-phosphate synthase (*ribB*) and riboflavin synthase (*ribC*), which improved 9α-OH-AD production and indicated that FAD availability affects sterol oxidation efficiency [18]. Su et al. improved phytosterol biotransformation by regulating the NAD^+^/NADH ratio, further highlighting the importance of NADH reoxidation in maintaining high-flux sterol oxidation [19]. In addition, the dense and hydrophobic cell envelope of *M. neoaurum* represents another major barrier to phytosterol uptake and steroid export. The mycobacterial cell envelope contains mycolic acids, trehalose monomycolate/trehalose dimycolate (TMM/TDM), lipomannan (LM), and lipoarabinomannan (LAM), which together form a low-permeability hydrophobic structure [21,22]. Previous studies have shown that deletion of arabinosyltransferase EmbC (*embC*), combined deletion of fibronectin-binding protein C3 (*fbpC3*) and *embC*, or disruption of β-ketoacyl-acyl carrier protein synthase B (*kasB*) can improve cell permeability and steroid conversion efficiency [23,24]. However, cell envelope engineering must be carefully controlled because excessive perturbation of cell wall architecture may impair cell morphology and growth stability. For example, simultaneous disruption of succinyltransferase (*sucT*) and trehalose monomycolate acyltransferase (*tmaT*) did not further improve 9α-OH-AD production, suggesting that excessive cell envelope perturbation may be unfavorable for stable biotransformation [25]. Therefore, efficient AD production requires not only metabolic flux rerouting but also balanced optimization of non-pathway factors, including substrate availability, cofactor regeneration, and cell permeability.

In this study, *M. neoaurum* LY-2 was employed as the microbial chassis to construct a high-performance platform for AD production from phytosterols. Initially, the phytosterol catabolic network of *M. neoaurum* LY-2 was systematically redirected by blocking competing C19 and C22 steroid branches, thereby establishing an AD-accumulating chassis strain. Subsequently, transcriptional regulation and central metabolic reinforcement were introduced to enhance sterol side-chain degradation and improve the utilization of acetyl-CoA and propionyl-CoA generated during phytosterol catabolism. To sustain intensified sterol oxidation, cofactor supply, NAD^+^ regeneration, and oxidative stress resistance were further optimized to maintain intracellular redox homeostasis. Finally, moderate cell envelope remodeling was performed to improve phytosterol uptake and promote AD accumulation without severely impairing cell growth. The final engineered strain was evaluated in a 5 L fermenter under high-substrate-loading conditions. This work provides a systematic and transferable strategy for constructing robust mycobacterial cell factories for steroid intermediate production.

## 2 Materials and methods

### 2.1 Chemicals and reagents

The reagents used in this study were sourced from Sinopharm (Shanghai, China), unless otherwise specified. Phytosterols (β-sitosterol 40.50%, Campesterol 23.82%, Stigmasterol 24.96%, Brassicasterol 1.07%) were purchased from Qionghua Biotech (Shaanxi, China). The Standards of AD, ADD, 9α-OH-AD, and 4-HBC were purchased from Aladdin (Shanghai, China). The 2 × Phanta Max Master Mix (Dye Plus) and one Step Cloning Kit were purchased from Vazyme Biotech (Nanjing, China). All restriction enzymes and T4 DNA Ligase were purchased from Takara (Dalian, China).

### 2.2 Strains, plasmids, and culture conditions

All strains and plasmids used in this study are listed in Tables S1 and S2, respectively, and all primers are listed in Table S3. *Escherichia coli* was used for routine plasmid construction and propagation, and *M*. *neoaurum* LY-2 was used as the parental strain for AD biosynthesis. Engineered strains were constructed stepwise according to the order of pathway modification and named Mbn1-Mbn30. Unless otherwise stated, *E. coli* strains were cultivated in Luria-Bertani medium at 37°C, while *M. neoaurum* strains were cultivated at 30°C with shaking. When required, antibiotics were added at appropriate concentrations for plasmid maintenance or strain selection.

The seed medium for *M. neoaurum* LY-2 contained glycerol 2 g/L, diammonium hydrogen phosphate 0.6 g/L, sodium nitrate 5.4 g/L, and yeast extract 15 g/L. For shake-flask biotransformation, *M. neoaurum* strains were first inoculated into the seed medium and cultivated to the exponential growth phase. The seed culture was then transferred into fermentation medium containing phytosterols as the substrate. The fermentation medium contained phytosterols 10 g/L, glucose 15 g/L, yeast extract 10 g/L, ammonium ferric citrate 0.05 g/L, MgSO_4_ 0.25 g/L, and (NH_4_)_2_HPO_4_ 0.15 g/L. Substrate solubilization was achieved using hydroxypropyl-β-cyclodextrin and soybean oil at a molar ratio of 1:2. Shake-flask biotransformation was carried out at 30°C with shaking for 192 h. Samples were collected at defined time points to determine cell growth, residual phytosterols, steroid products, and intracellular physiological parameters.

### 2.3 Genome mining and phylogenetic analysis of KstD homologs

The genome sequence of *M. neoaurum* LY-2 was used to identify candidate genes involved in steroid catabolism. Putative 3-ketosteroid-Δ^1^-dehydrogenase genes were identified by local sequence similarity searches using reported KstD proteins from mycobacteria and related actinobacteria as queries. Conserved domains were analyzed using public protein-domain databases. The amino acid sequences of KstD homologs were aligned, and a phylogenetic tree was constructed to evaluate their evolutionary relationships. The resulting analysis was used to guide subsequent functional characterization of the KstD isoenzymes.

### 2.4 Genetic manipulation

Targeted gene deletion and chromosomal overexpression in *M. neoaurum* LY-2 were performed using a p2NILB-based homologous recombination strategy. The p2NILB plasmid, derived from p2NIL and carrying the SacB counter-selection marker, was used as the backbone for constructing deletion and integration vectors. For gene deletion, approximately 1000 bp upstream and downstream homologous arms flanking each target gene were amplified from the LY-2 genome and assembled into p2NILB to generate the corresponding deletion plasmids, such as p2NILB-KstD1UD, p2NILB-KshA1UD, p2NILB-SalAUD, p2NILB-FdmRUD, p2NILB-KasBUD, p2NILB-FbpC3UD, and p2NILB-EmbCUD. The recombinant plasmids were introduced into *M. neoaurum* by electroporation. Candidate mutants were screened by antibiotic selection followed by sacB-mediated sucrose counter-selection, and correct allelic exchange events were verified by colony PCR using primers flanking the homologous arms. The PCR products were further confirmed by Sanger sequencing.

For chromosomal overexpression, the coding sequences of target genes were assembled into expression cassettes and inserted into selected chromosomal loci using p2NILB-derived integration plasmids. For example, p2NILB-KshA1UD-Icl1 and p2NILB-KshA2UD-PrpR were constructed to insert the *icl1* and *prpR* expression cassettes into the 3-ketosteroid-9α-hydroxylase oxygenase component A1 (*kshA1*) and 3-ketosteroid-9α-hydroxylase oxygenase component A2 (*kshA2*) loci, respectively. Similarly, p2NILB-derived integration plasmids carrying GTP cyclohydrolase II (*ribA*), 3,4-dihydroxy-2-butanone 4-phosphate synthase (*ribB*), riboflavin synthase (*ribC*), 5-amino-6-(5-phosphoribosylamino)uracil reductase/deaminase (*ribD*), riboflavin kinase/FMN adenylyltransferase (*ribF*), 6,7-dimethyl-8-ribityllumazine synthase (*ribH*), type II NADH dehydrogenase (*ndhF*), and catalase (*katA*) expression cassettes were constructed for enhancing FAD biosynthesis, NAD^+^ regeneration, and oxidative stress resistance. The presence of the inserted expression cassettes in the engineered strains was confirmed by colony PCR and Sanger sequencing. All recombinant plasmids and primers used in this study are listed in Tables S2 and S3, respectively.

Using this strategy, five 3-ketosteroid-Δ^1^-dehydrogenase genes (*kstD1*–*kstD5*) were deleted individually and in combination to block steroid Δ^1^-dehydrogenation, while *kshA1* and *kshA2* were deleted to attenuate 9α-hydroxylation. The C22 branch was further weakened by deletion of *salA* and *opccR*. For transcriptional regulation, *fdmR* and 3-ketosteroid 9α-hydroxylase regulator (*kstR*) were selected as candidate regulatory targets, and *fdmR* deletion was used for subsequent strain construction. To improve central metabolic capacity, *icl1* and *prpR* were overexpressed to reinforce the glyoxylate and methylcitrate cycles. To improve redox homeostasis, *ribB*/*ribC*, *ndhF*, and *katA* were overexpressed to enhance FAD supply, NAD^+^ regeneration, and H_2_O_2_ detoxification, respectively. For cell envelope remodeling, *kasB*, *fbpC3*, and *embC* were deleted individually or in combination to evaluate their effects on cell permeability and AD production.

### 2.5 RNA extraction and qRT-PCR analysis

Cells were harvested during phytosterol biotransformation for transcriptional analysis. Cells were harvested after 48 h of cultivation. Total RNA was extracted using the HiScript II RT SuperMix Kit (Vazyme, China) in combination with a FastPrep homogenizer according to the manufacturer's instructions. After genomic DNA removal, first-strand cDNA was synthesized from 1 μg of total RNA using HiScript II qRT SuperMix. Quantitative real-time PCR (qRT-PCR) was performed using Chem Q™ SYBR Green Master Mix on a real-time PCR system. Each 20 μL reaction mixture contained 10 μL of 2× SYBR mix, 0.4 μL of each primer, and 50 ng of cDNA template. The thermal cycling program consisted of an initial denaturation at 95°C for 10 min, followed by 40 cycles of 95°C for 15 s, 58°C for 15 s, and 72°C for 30 s. Melting curve analysis was performed to confirm amplification specificity. Gene expression levels were normalized to the internal reference gene 16S rRNA, and relative expression levels were calculated using the 2^-ΔΔCT method [26,27]. Primers used for qRT-PCR are listed in Table S3.

### 2.6 Determination of intracellular metabolites and oxidative stress indicators

Intracellular metabolites and oxidative stress indicators were determined to evaluate metabolic pressure and redox status during phytosterol conversion. Strain biomass was expressed as dry cell weight (DCW), and DCW was determined as previously described [28]. Cell samples were collected at defined time points, rapidly harvested, washed, and used for metabolite extraction or physiological analysis.

Intracellular acetyl-CoA concentration was measured using a coupled enzyme assay with minor modifications [14]. Briefly, cell extracts were deproteinized with 6% perchloric acid and neutralized with KOH. Then, 10 μL of the treated sample was mixed with 50 μL of reaction mixture in a 96-well plate. After incubation at 37°C for 10 min in the dark, fluorescence intensity was measured using a microplate reader at an excitation wavelength of 535 nm and an emission wavelength of 587 nm. Blank wells were used for background correction. Intracellular propionyl-CoA concentration was determined according to the previously reported method [28]. ATP content was measured as described previously [19]. FAD, NAD^+^, and NADH levels were determined using commercial assay kits or chromatographic methods according to the manufacturers’ instructions. The NAD^+^/NADH ratio was calculated from the measured NAD^+^ and NADH concentrations. All intracellular metabolite levels were normalized to DCW when appropriate.

Reactive oxygen species (ROS) levels and extracellular hydrogen peroxide (H_2_O_2_) accumulation were measured to evaluate oxidative stress. Intracellular ROS levels were determined using a fluorescent probe according to a previously reported method [29]. The cells were incubated with the probe under dark conditions and then analyzed using a fluorescence spectrophotometer or microplate reader. Extracellular H_2_O_2_ concentration was determined using a commercial H_2_O_2_ assay kit according to the protocol provided by the manufacturer. The measured ROS and H_2_O_2_ values were normalized to cell biomass or DCW when appropriate.

### 2.7 Determination of cell wall permeability

Cell wall permeability was evaluated by measuring the fluorescence intensity of cells stained with fluorescein diacetate (FDA), following a previously reported method with minor modifications. The engineered strains were cultivated under identical conditions, harvested at the same growth phase, and normalized based on wet cell weight. Strain Mbn26, the parental strain before cell envelope remodeling, was used as the control. Cells were resuspended in 4.5 mL of phosphate buffer to achieve a final cell density corresponding to an OD600 of 0.4–0.5. Subsequently, 0.5 mL of FDA solution (2 mg/mL in acetone) was added to the cell suspension, and the mixture was incubated at 30°C with gentle shaking for 30 min to allow intracellular esterase-mediated hydrolysis of FDA. A blank sample without FDA was included to correct background fluorescence. After incubation, the fluorescence intensity of the stained cells was measured using a fluorescence spectrophotometer. The excitation and emission wavelengths were set to 485 nm and 538 nm, respectively. Cell wall permeability was expressed as the relative fluorescence fold change compared with Mbn26. Increased fluorescence intensity was interpreted as enhanced cell wall permeability.

### 2.8 5 L fermenter biotransformation

Scale-up phytosterol biotransformation was carried out in a 5 L fermenter to evaluate the production performance of Mbn12 and the final engineered strain Mbn30. The fermentation medium contained phytosterols 30 g/L, glucose 15 g/L, glycerin 10 g/L, yeast extract 20 g/L, ammonium ferric citrate 0.1 g/L, MgSO_4_ 0.25 g/L, and (NH_4_)_2_HPO_4_ 0.15 g/L. Substrate solubilization was still performed using 2-hydroxypropyl-β-cyclodextrin and soybean oil. Fermentation was conducted at 30°C for 192 h, and the pH was maintained at 7.0 throughout the process by automatic addition of acid or alkali. Samples were collected periodically to determine glucose consumption, OD_600_, residual phytosterols, and AD accumulation. The performance of Mbn30 was compared with that of Mbn12, the basal strain capable of accumulating AD, to evaluate the effect of integrated metabolic engineering under controlled fermentation conditions.

### 2.9 Extraction and analysis methods

During phytosterol biotransformation by *M*. *neoaurum* LY-2 and its engineered derivatives, fermentation broth samples were collected at defined time intervals. For steroid extraction, 1 mL of fermentation broth was extracted three times with an equal volume of ethyl acetate. After vigorous mixing and centrifugation, the organic phase was collected and combined. The organic extract was evaporated to dryness, and the residue was dissolved in 4.0 mL of methanol. The resulting solution was filtered through a 0.22 μm membrane before high-performance liquid chromatography (HPLC) analysis.

Steroid products were analyzed using an HPLC system equipped with an SDB C18 column (5 μm, 4.6 × 250 mm; Elite, China) maintained at 30°C. Methanol/water (8:2, v/v) was used as the mobile phase at a flow rate of 1.0 mL/min. AD and other steroid intermediates were detected by UV absorbance at 254 nm. Product identities were confirmed by comparison with authentic standards, and AD concentration was calculated using the corresponding standard curve. Glucose and residual phytosterols were analyzed using suitable chromatographic or colorimetric methods.

### 2.10 Statistical analysis

All experiments were performed in triplicate, and data are presented as the mean ± standard deviation (SD). Unless otherwise stated, statistical analysis was performed using one-way analysis of variance (one-way ANOVA). Differences were considered statistically significant at P < 0.05. Significance levels are indicated in the figures as follows: *P < 0.05, **P < 0.01, and ***P < 0.001. Figures were generated using Origin software version 10.1.

## 3 Results and discussion

### 3.1 Rerouting C19 and C22 steroid catabolic pathways enables selective AD accumulation

**Fig. 1:**
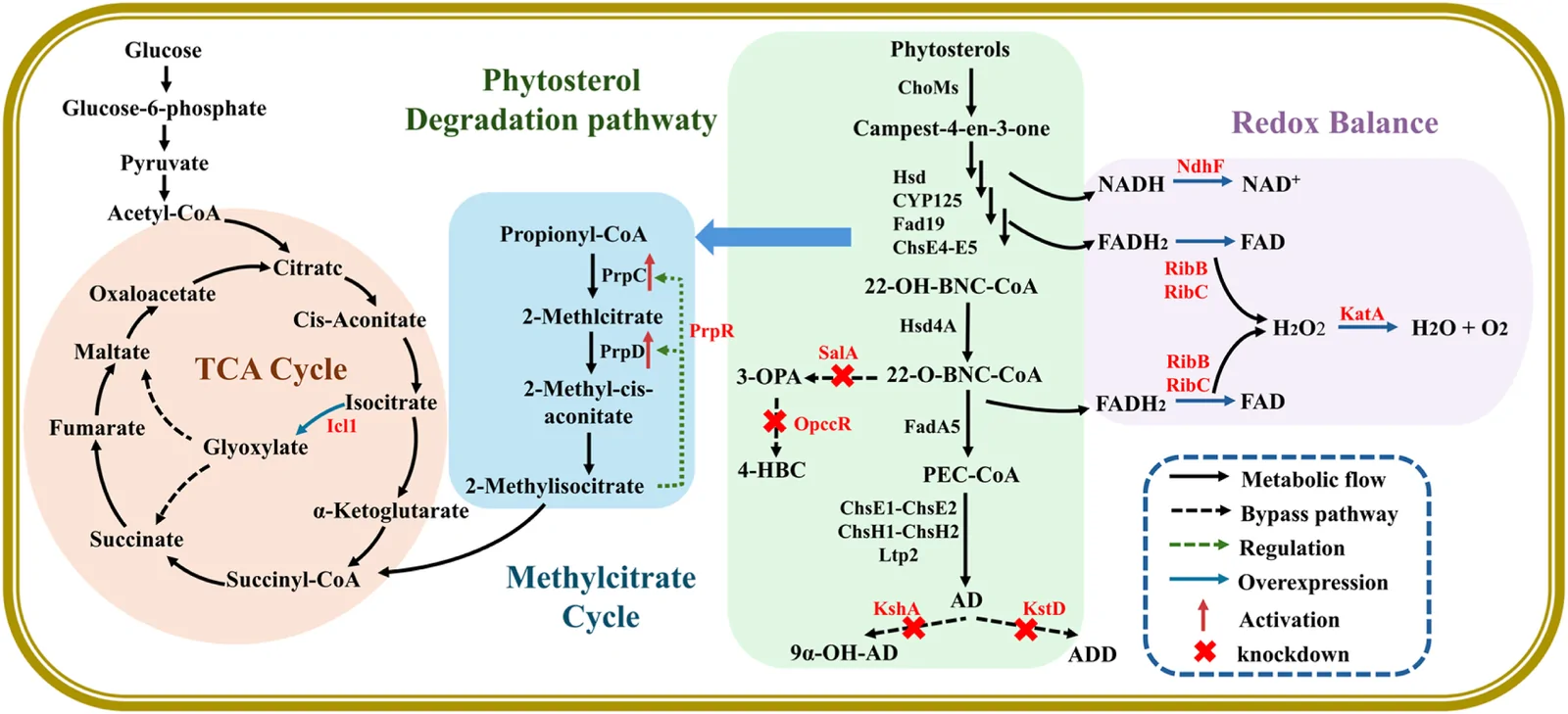
Schematic overview of the metabolic engineering strategy for directing phytosterol catabolism toward androstenedione production in *M*. *neoaurum* LY-2. Phytosterol degradation generates AD through sterol nucleus oxidation and side-chain degradation, accompanied by the release of acetyl-CoA and propionyl-CoA, which are further assimilated through the TCA cycle and methylcitrate cycle. Competing steroid branches were blocked by suppressing *kstD*, *kshA*, *salA*, and *opccR* to reduce ADD, 9α-OH-AD, and 4-HBC formation. Solid arrows indicate metabolic flow, dashed arrows indicate bypass pathways, green dashed arrows indicate regulation, blue arrows indicate overexpression, upward red arrows indicate activation, and red crosses indicate gene knockdown.

**Fig. 2:**
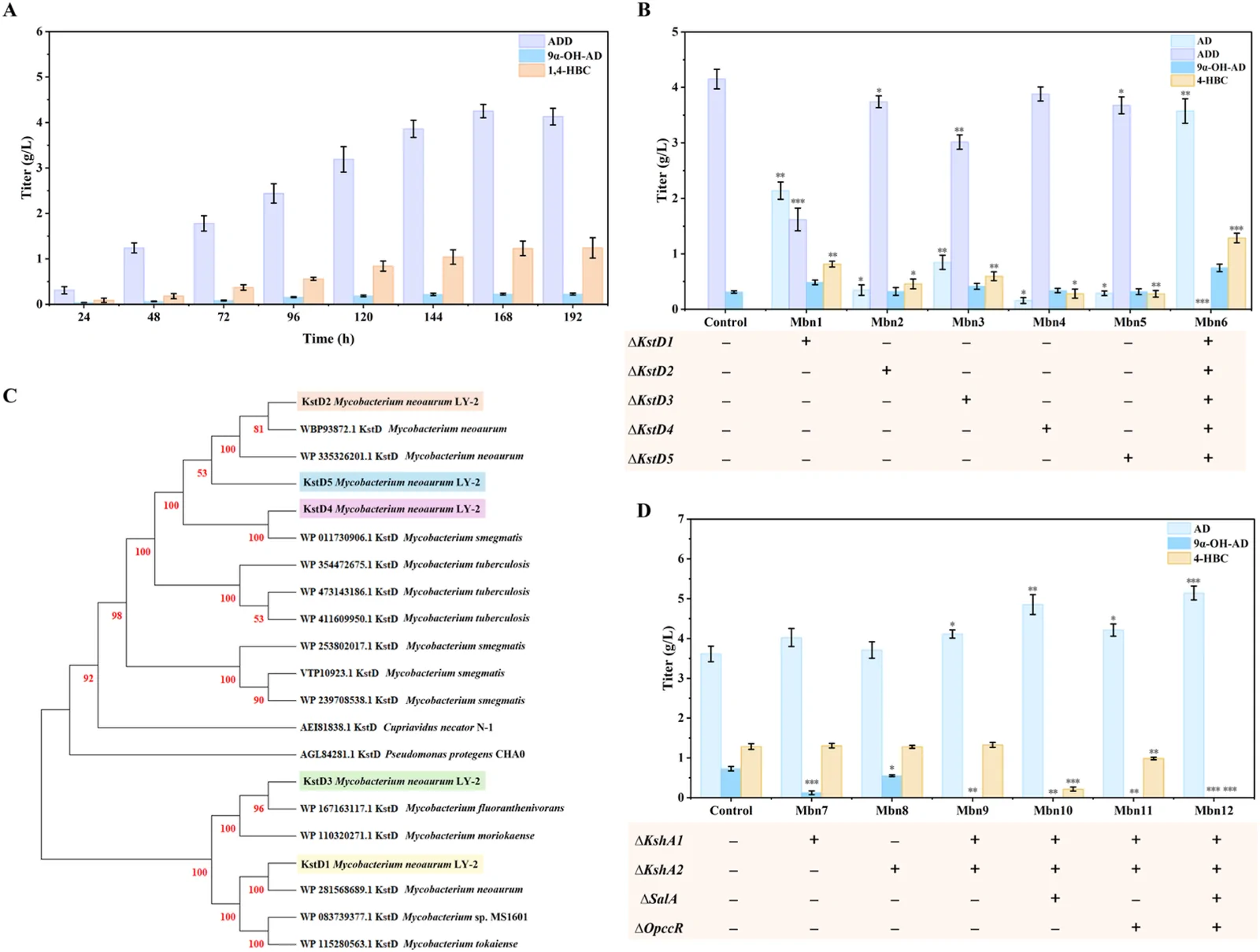
Rerouting C19 and C22 steroid catabolic pathways for selective AD accumulation in *M*. *neoaurum* LY-2. (A) Time-course analysis of phytosterol bioconversion by the parental strain LY-2. ADD, 9α-OH-AD, and 1,4-HBC were monitored during transformation. (B) Effects of single and combined *kstD* deletions on steroid product distribution. (C) Phylogenetic analysis of KstD homologs from LY-2 and representative bacterial strains. The KstD1-5 from LY-2 are highlighted in different colors. (D) Effects of deleting key genes involved in the C22 pathway and alternative C19 pathways on AD accumulation and by-product formation.

Phytosterol degradation in *M*. *neoaurum* proceeds through the C19 and C22 steroid catabolic branches, generating ADD, 9α-OH-AD, 1,4-HBC and other intermediates [30](Fig. 1). Because AD is an intermediate rather than an endpoint in the native sterol catabolic network, its accumulation depends on simultaneously preventing downstream C19 conversion and reducing diversion into the C22 branch. To characterize the conversion profile of *M. neoaurum* LY-2, phytosterols were used as substrates in a time-course biotransformation. LY-2 mainly accumulated ADD, which increased continuously and reached 4.25 g/L at 168 h (Fig. 2A). The C22 by-product 1,4-HBC also accumulated gradually to 1.23 g/L, whereas 9α-OH-AD remained low, with a maximum titer of 0.22 g/L. AD was barely detected throughout the process, indicating that the generated AD was rapidly converted to downstream products. Thus, although LY-2 showed strong phytosterol-degrading ability, its metabolic flux was mainly directed toward ADD and C22 by-products rather than AD.

To attenuate AD degradation in the C19 branch, Δ^1^-dehydrogenation catalyzed by 3-ketosteroid-Δ^1^-dehydrogenase and 9α-hydroxylation catalyzed by 3-ketosteroid-9α-hydroxylase were targeted [11,12]. Five *kstD* genes were identified in LY-2 by whole-genome sequencing. Phylogenetic analysis showed that the encoded KstD proteins clustered with reported KstD homologs from *M. neoaurum*, *M. smegmatis*, and *M. tuberculosis*, suggesting their potential involvement in steroid Δ^1^-dehydrogenation (Fig. 2C). To evaluate the contribution of each KstD isoenzyme to ADD formation, single *kstD* deletion mutants were constructed. In Mbn1, deletion of *kstD1* reduced ADD from 4.15 to 1.62 g/L and increased AD accumulation to 2.14 g/L, indicating that KstD1 is the major Δ^1^-dehydrogenase responsible for the conversion of AD to ADD (Fig. 2B). In contrast, deletion of *kstD2*, *kstD4* or *kstD5* resulted in only low AD accumulation, reaching 0.35, 0.16 and 0.29 g/L, respectively. The deletion of *kstD3* increased AD to 0.85 g/L, suggesting a minor role in Δ^1^-dehydrogenation. These isoenzyme-specific differences suggest that the five KstD proteins are not functionally equivalent in LY-2, and that AD dehydrogenation is mainly carried by KstD1 with partial support from KstD3. The quintuple *kstD* deletion strain Mbn6 was then constructed to eliminate residual Δ^1^-dehydrogenase activity. ADD was no longer detected in Mbn6, whereas AD production increased to 3.58 g/L. This result confirms that complete removal of KstD activity effectively prevents the principal C19 route of AD degradation. Meanwhile, the C22 product profile also altered. The parental strain accumulated 1,4-HBC, whereas only 1.33 g/L 4-HBC was detected in Mbn6. This shift is mechanistically consistent with the ability of KstD enzymes to catalyze Δ^1^-dehydrogenation not only in C19 steroids but also in C22 intermediates, thereby converting 4-HBC to 1,4-HBC. However, Mbn6 still accumulated 0.75 g/L 9α-OH-AD, indicating that part of the AD pool entered the 9α-hydroxylation pathway after Δ^1^-dehydrogenation was blocked. The parental strain accumulated 1,4-HBC, whereas only 1.33 g/L 4-HBC was detected in Mbn6. This shift is mechanistically consistent with the ability of KstD enzymes to catalyze Δ^1^-dehydrogenation not only in C19 steroids but also in C22 intermediates, thereby converting 4-HBC to 1,4-HBC. However, Mbn6 still accumulated 0.75 g/L 9α-OH-AD, indicating that part of the AD pool entered the 9α-hydroxylation pathway after Δ^1^-dehydrogenation was blocked.

KshA is the oxygenase component of 3-ketosteroid-9α-hydroxylase and usually cooperates with the reductase component KshB to catalyze steroid nucleus 9α-hydroxylation [31]. Genome analysis revealed two putative *kshA* genes in LY-2, *kshA1* and *kshA2*. To determine their contributions to 9α-OH-AD formation, *kshA1* and *kshA2* deletion mutants were constructed from Mbn6. The deletion of *kshA1* in Mbn7 reduced 9α-OH-AD from 0.73 to 0.12 g/L and increased AD production to 4.02 g/L (Fig. 2D). In contrast, deletion of *kshA2* in Mbn8 still allowed 0.55 g/L 9α-OH-AD accumulation, with an AD titer of 3.71 g/L. When *kshA1* and *kshA2* were simultaneously deleted, 9α-OH-AD was no longer detected in Mbn9, and AD production increased to 4.12 g/L. These results indicate that KshA1 is the major component responsible for 9α-hydroxylation in LY-2, whereas simultaneous deletion of *kshA1* and *kshA2* more completely blocked hydroxylated by-product formation. After eliminating ADD and 9α-OH-AD formation in the C19 branch, the C22 branch could still compete for phytosterol-derived metabolic flux. Previous studies have shown that SalA is a steroid aldolase involved in C24 steroid side-chain cleavage and plays an important role in HBC-type C22 intermediate formation [13]. OpccR is a 3-oxo-4-pregnene-20-carboxyl-CoA reductase involved in the formation of 4-HBC-type products in the C22 branch [12]. Therefore, *salA* and *opccR* were further deleted from Mbn9 to attenuate the C22 branch. The deletion of *salA* reduced 4-HBC from 1.33 to 0.22 g/L and increased AD production to 4.85 g/L, indicating that SalA was the major contributor to C22 by-product formation (Fig. 2D). The deletion of *opccR* reduced 4-HBC to 0.99 g/L and resulted in 4.21 g/L AD, suggesting that OpccR also contributed to C22 product formation but had a weaker effect than SalA. Further simultaneous deletion of *salA* and *opccR* eliminated both 4-HBC and 9α-OH-AD in Mbn12 and increased AD production to 5.14 g/L (Fig. 2D). These results indicate that SalA is the major entry point for C22 by-product formation, whereas OpccR contributes to downstream C22 product generation. By sequentially eliminating AD-consuming reactions and the competing C22 branch, LY-2 was converted from an ADD-producing strain into a basal AD-accumulating chassis.

### 3.2 Transcriptional regulation enhances phytosterol catabolic flux

Although Mbn12 efficiently directed phytosterol-derived metabolic flux toward AD accumulation, further improvement of AD production was still constrained by upstream substrate uptake, initial oxidation of the steroid nucleus, and stepwise degradation of the sterol side chain. Phytosterol side-chain degradation involves multiple CoA activation and β-oxidation-like reactions, which represent critical steps controlling the flux of substrate into the AD biosynthetic route [8,9]. Thus, after downstream AD degradation was blocked, the next engineering objective was to increase the productive upstream flux feeding AD biosynthesis rather than further modifying the AD endpoint itself.

**Fig. 3:**
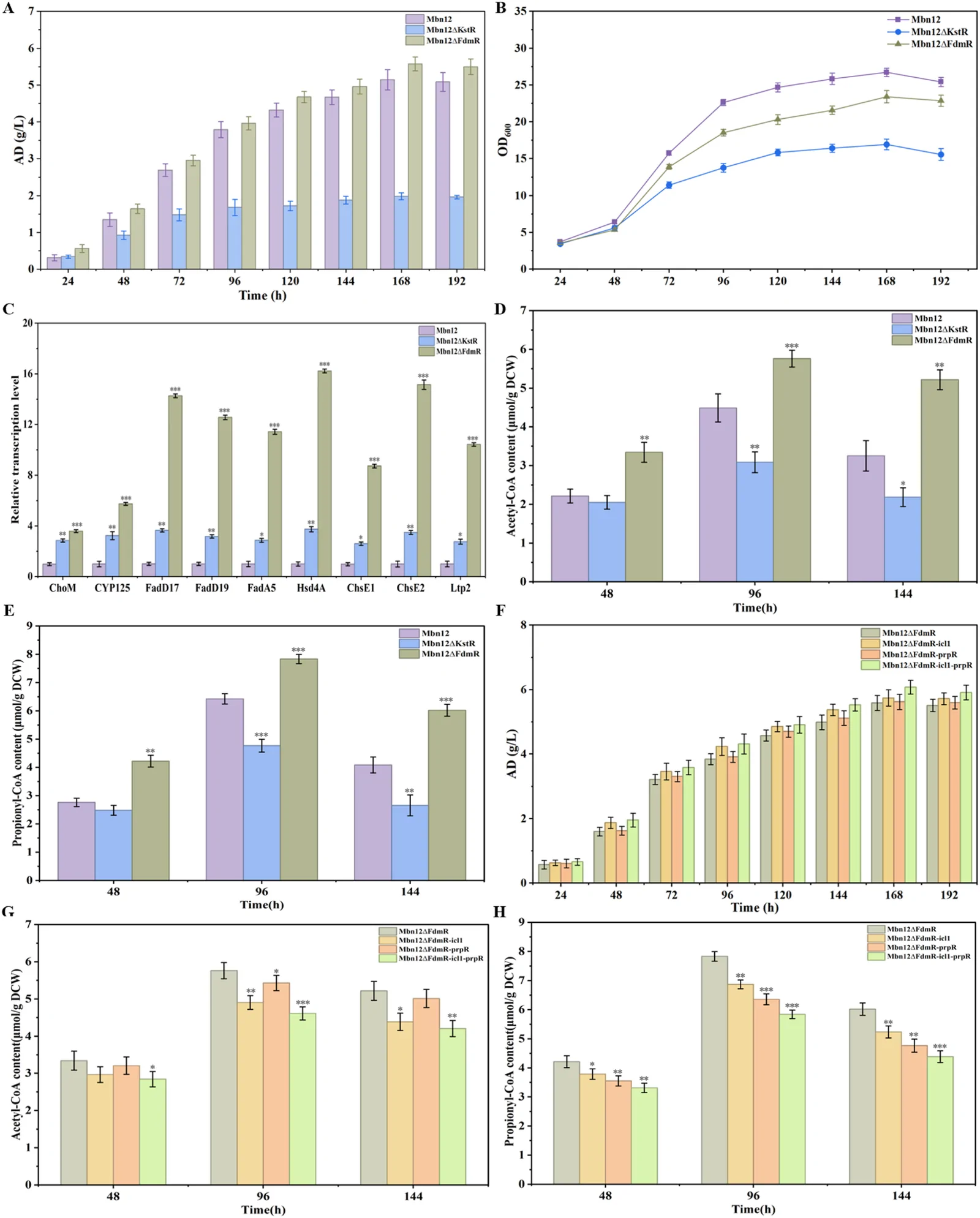
Transcriptional regulation enhances phytosterol catabolic flux. (A) The AD production of Mbn12, Mbn12ΔKstR, and Mbn12ΔFdmR during phytosterol conversion. (B) Intracellular acetyl-CoA levels in Mbn12, Mbn12ΔKstR, and Mbn12ΔFdmR. (C) Relative transcription levels of key genes involved in phytosterol side-chain degradation and steroid intermediate conversion. (D) Intracellular propionyl-CoA levels in Mbn12, Mbn12ΔKstR, and Mbn12ΔFdmR. (E) The AD production of Mbn12ΔFdmR-derived strains. (F) Growth profiles of Mbn12ΔFdmR-derived strains. (G) Intracellular acetyl-CoA levels in Mbn12ΔFdmR-derived strains. (H) Intracellular propionyl-CoA levels in Mbn12ΔFdmR-derived strains.

Two TetR-family transcriptional regulators associated with mycobacterial sterol metabolism, FdmR and KstR, were selected and compared. FdmR is mainly associated with fatty acid metabolism and sterol side-chain degradation [14], whereas KstR broadly controls genes related to sterol uptake, initial oxidation, side-chain degradation, and downstream steroid nucleus catabolism [32]. This difference in regulatory range provided a clear engineering rationale. FdmR deletion was expected to release repression on side-chain degradation genes and enhance precursor supply for AD formation, whereas KstR deletion could activate a broader sterol regulon and potentially increase nonproductive metabolism or physiological burden. Compared with Mbn12, Mbn12ΔFdmR showed enhanced AD accumulation throughout fermentation, reaching 5.50 g/L at 192 h, compared with 5.09 g/L for Mbn12 (Fig. 3A). In contrast, Mbn12ΔKstR produced only 1.96 g/L AD at 192 h and showed impaired growth, with an OD600 of 15.58 compared with 25.43 for Mbn12 and 22.85 for Mbn12ΔFdmR (Fig. S1A). Therefore, global derepression by KstR deletion did not translate into productive AD flux, while the more focused FdmR perturbation improved AD formation with limited loss of physiological robustness.

RT-qPCR analysis further revealed the molecular basis underlying the distinct phenotypes of Mbn12ΔFdmR and Mbn12ΔKstR. The selected genes represented key steps in phytosterol catabolism, including sterol uptake and initial oxidation, side-chain activation, β-oxidation-like degradation, and steroid intermediate conversion. Specifically, *choM* (encoding a cholesterol oxidase/3β-hydroxysteroid dehydrogenase-like enzyme) and *cyp125* (encoding cytochrome P450 125) are associated with the initial oxidation of sterol substrates. The *fadD17* and *fadD19* encode acyl-CoA synthetase-related enzymes involved in side-chain activation. The *fadA5* (encoding acetyl-CoA acetyltransferase/thiolase) and *hsd4A* (encoding a 17β-hydroxysteroid dehydrogenase/3β-hydroxyacyl-CoA dehydrogenase-like enzyme) participate in β-oxidation-like side-chain degradation. The *chsE1* and *chsE2* encode acyl-CoA dehydrogenase subunits, and *ltp2* (encoding a lipid transfer protein) is associated with steroid side-chain degradation and intermediate conversion. These genes were therefore used as representative markers to determine whether regulator deletion enhanced the transcriptional activity of the sterol catabolic module. In Mbn12ΔFdmR, these genes were significantly upregulated (Fig. 3C), indicating that release of FdmR-mediated repression strengthened the side-chain degradation module. In Mbn12ΔFdmR, these genes were significantly upregulated (Fig. 3C), indicating that release of FdmR-mediated repression strengthened the side-chain degradation module. This difference was further supported by intracellular acyl-CoA and ATP profiles. Mbn12ΔFdmR maintained higher acetyl-CoA and propionyl-CoA levels at 48, 96, and 144 h, with maximum values of 5.76 and 7.83 μmol/g DCW at 96 h, respectively (Fig. 3B and D). It also showed a higher ATP level at 48 and 96 h, reaching approximately 6.90 μmol/g DCW at 96 h (Fig. S1B). Mbn12ΔKstR showed lower acetyl-CoA, propionyl-CoA, and ATP levels, particularly at 96 and 144 h. Its ATP level decreased to approximately 4.06 μmol/g DCW at 96 h and 1.84 μmol/g DCW at 144 h. These results suggest that FdmR deletion increased the generation of C2 and C3 units from side-chain degradation while preserving energy supply. In contrast, KstR deletion may have broadly activated sterol metabolism and increased CoA and ATP demand, thereby weakening central metabolic capacity and preventing the upregulated pathway from sustaining AD production [33].

To further alleviate the acyl-CoA accumulation pressure caused by enhanced side-chain degradation after *fdmR* deletion, and *icl1* and *prpR* were selected as central metabolic engineering targets. The *icl1* encodes isocitrate lyase, a key enzyme of the glyoxylate cycle that facilitates the entry of acetyl-CoA into central carbon metabolism. Meanwhile, The Icl1 can also function as methylisocitrate lyase in the methylcitrate cycle, thereby participating in propionyl-CoA metabolism [15,34]. The PrpR is a transcriptional regulator associated with propionate and propionyl-CoA metabolism and can activate the expression of genes involved in the methylcitrate cycle, promoting further conversion of propionyl-CoA [35]. Thus, co-engineering *icl1* and *prpR* was designed to match the increased production of acetyl-CoA and propionyl-CoA with an improved capacity to assimilate these C2 and C3 units. Overexpression of *icl1* decreased acetyl-CoA and propionyl-CoA levels to 4.91 and 6.87 μmol/g DCW at 96 h, respectively (Fig. 3G and H). The *prpR* overexpression had a stronger effect on propionyl-CoA, decreasing it to 6.36 μmol/g DCW. Co-expression of *icl1* and *prpR* further reduced acetyl-CoA and propionyl-CoA to 4.61 and 5.84 μmol/g DCW, respectively. The resulting strain Mbn17 reached an OD600 of approximately 25.2 at 168 h and achieved the highest AD titer of approximately 6.08 g/L (Fig. 3E and F). Therefore, reinforcing the glyoxylate and methylcitrate cycles converted the benefit of FdmR-mediated side-chain activation into a balanced AD-producing flux by reducing acyl-CoA accumulation and improving central metabolic homeostasis.

### 3.3 Coordinated regeneration of FAD and NAD ^+^ improves intracellular redox homeostasis

**Fig. 4:**
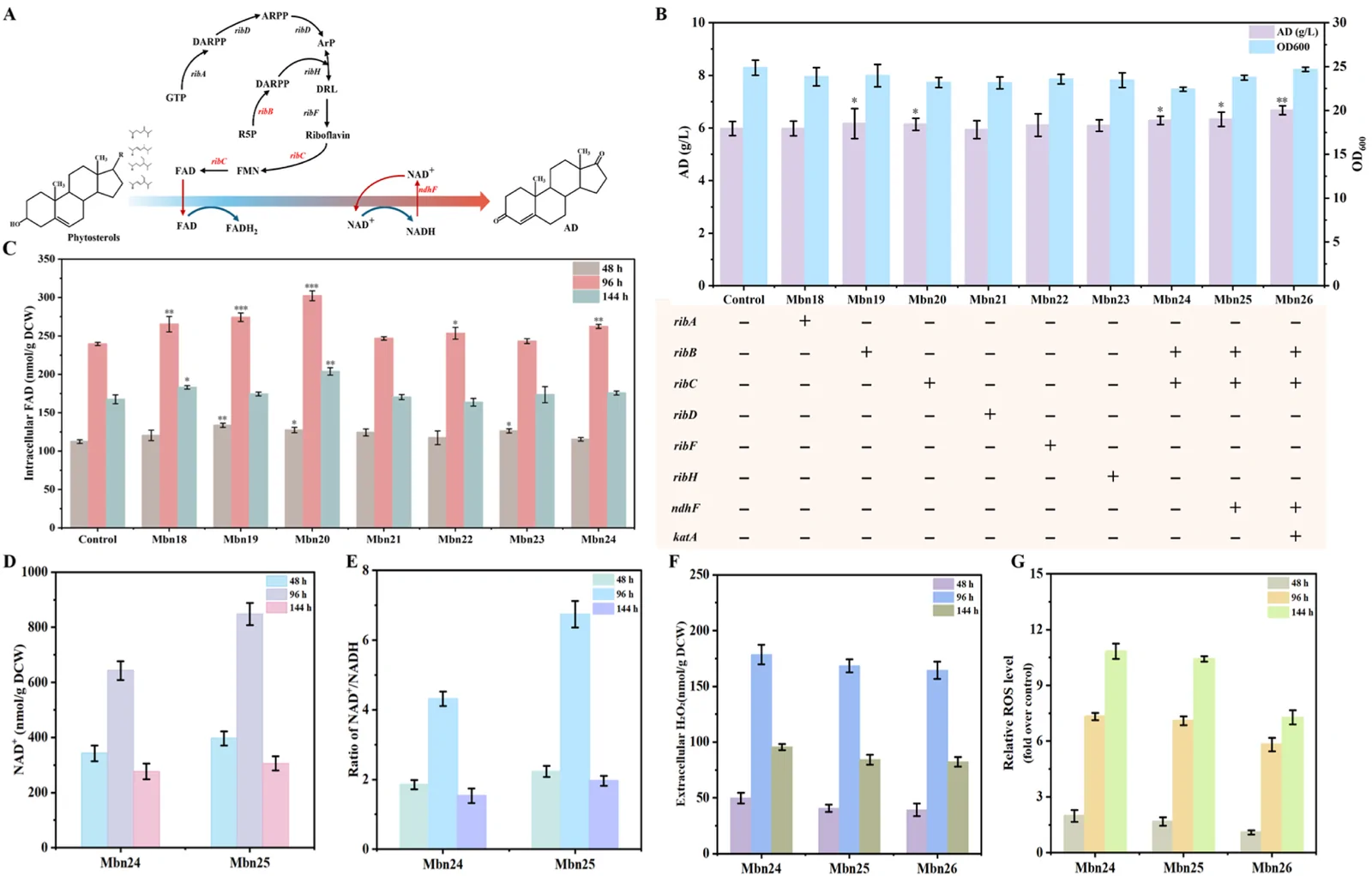
Coordinated regeneration of FAD and NAD ^+^ improves intracellular redox homeostasis during phytosterol bioconversion to AD. (A) Schematic representation of FAD biosynthesis, NAD^+^ regeneration, and oxidative-stress relief during phytosterol conversion to AD. (B) The effect of overexpressing different genes on AD production and cell growth. (C) Intracellular FAD levels of strains overexpressing different FAD biosynthesis genes. (D) Intracellular NAD^+^ levels in engineered strains. (E) NAD^+^/NADH ratios in engineered strains. (F) Extracellular H_2_O_2_ accumulation in engineered strains. (G) Relative intracellular ROS levels in engineered strains.

Phytosterol side-chain degradation toward AD involves multiple FAD- and NAD^+^-dependent oxidative reactions, during which FAD and NAD ^+^ are reduced to FADH_2_ and NADH, respectively (Fig. 4A) [18,29,30]. Enhancing side-chain flux can therefore increase the demand for oxidized cofactors and intensify electron flow into the respiratory chain. If reduced cofactors are not efficiently reoxidized, redox imbalance and reactive oxygen species formation may occur. Consistently, Mbn17 showed higher ROS and H_2_O_2_ levels than Mbn12 at all tested time points. Extracellular H_2_O_2_ increased to 194.25 nmol/g DCW at 96 h, while the relative ROS level reached 11.34 at 144 h (Fig. S2A and S2B). These results indicate that the flux improvement obtained in Mbn17 created a new redox bottleneck, making coordinated cofactor regeneration and oxidative stress mitigation necessary for further AD improvement.

In microorganisms, FAD biosynthesis mainly uses GTP and R5P as precursors and proceeds through multiple enzymatic reactions encoded by genes such as *ribA*, *ribB*, *ribC*, *ribD*, *ribF*, and *ribH* (Fig. 4A) [36]. To increase intracellular FAD cofactor supply, these six FAD biosynthesis-related genes were individually overexpressed in Mbn17 to evaluate their effects on phytosterol-to-AD conversion. All single-gene overexpression strains showed increased AD production and intracellular FAD levels compared with the control, indicating that enhanced FAD biosynthesis facilitated sterol oxidation. Among them, the *ribB* and *ribC* overexpression showed more pronounced effects, increasing AD production to 6.17 and 6.14 g/L (Fig. 4B), respectively, compared with 5.98 g/L in the control strain. Intracellular FAD levels also increased markedly during the middle stage of fermentation, with the *ribC* overexpression strain reaching the highest value among the single-gene overexpression strains, 302.31 nmol/g DCW at 96 h (Fig. 4C). Based on these results, *ribB* and *ribC* were further co-overexpressed to construct strain Mbn24. Compared with the control strain, Mbn24 further increased AD production to 6.29 g/L, the highest among all tested strains (Fig. 4B). Meanwhile, the intracellular FAD level reached 262.40 nmol/g DCW at 96 h, which was higher than that of the control strain at 239.51 nmol/g DCW (Fig. 4C). However, although extracellular H_2_O_2_ and relative ROS levels decreased to some extent in the *ribB*, *ribC*, and *ribB*/*ribC* overexpression strains compared with Mbn17, they remained relatively high. In Mbn24, extracellular H_2_O_2_ and relative ROS levels at 96 h were still 178.54 nmol/g DCW and 7.32, respectively (Fig. S3A and S3B). Thus, increasing FAD biosynthesis improved oxidative conversion capacity but was insufficient to fully relieve redox pressure caused by restricted reduced-cofactor turnover [18].

To alleviate redox pressure during high-flux sterol conversion, the endogenous *ndhF* gene was overexpressed in Mbn24 to enhance NAD ^+^ regeneration. The *ndhF* encodes type II NADH dehydrogenase, which catalyzes NADH oxidation to NAD^+^ and transfers electrons to quinone electron acceptors in the respiratory chain (Fig. 4A) [29]. The AD production increased to 6.33 g/L in Mbn25, while the intracellular NAD^+^ level and NAD^+^/NADH ratio at 96 h increased to 847.65 nmol/g DCW and 6.74, respectively, indicating enhanced NADH reoxidation capacity (Fig. 4D and E). Meanwhile, extracellular H_2_O_2_ accumulation decreased in Mbn25. However, the relative ROS levels at 96 and 144 h still reached 7.10 and 10.42, respectively, suggesting that enhancing NAD^+^ regeneration alone was not sufficient to fully alleviate oxidative stress (Fig. 4F and G). Therefore, *katA*, encoding catalase, was overexpressed to enhance H_2_O_2_ detoxification [30]. In Mbn26, extracellular H_2_O_2_ at 96 h decreased from 168.39 to 164.32 nmol/g DCW, and the relative ROS level decreased from 7.10 to 5.82 (Fig. 4F and G). The AD production further increased to 6.67 g/L (Fig. 4B). Together, these results show that FAD supply, NAD^+^ regeneration, and H_2_O_2_ removal act at different points of the redox network. Their coordination improved intracellular redox homeostasis more effectively than any single intervention and supported sustained AD biosynthesis.

### 3.4 Enhancing cell permeability facilitates androstenedione biosynthesis from phytosterols

Phytosterols are highly hydrophobic substrates, and their transport into *M*. *neoaurum* cells is strongly restricted by the cell envelope barrier. The cell envelope of *M. neoaurum* is rich in mycolic acids and their derivative lipid components, including trehalose monomycolate (TMM), trehalose dimycolate (TDM), lipomannan (LM), and lipoarabinomannan (LAM) [22,37]. After pathway flux and redox balance had been improved, substrate transfer across this hydrophobic envelope became a likely physical bottleneck. Therefore, cell envelope remodeling was introduced to improve substrate uptake and product release without severely compromising cell integrity.

**Fig. 5:**
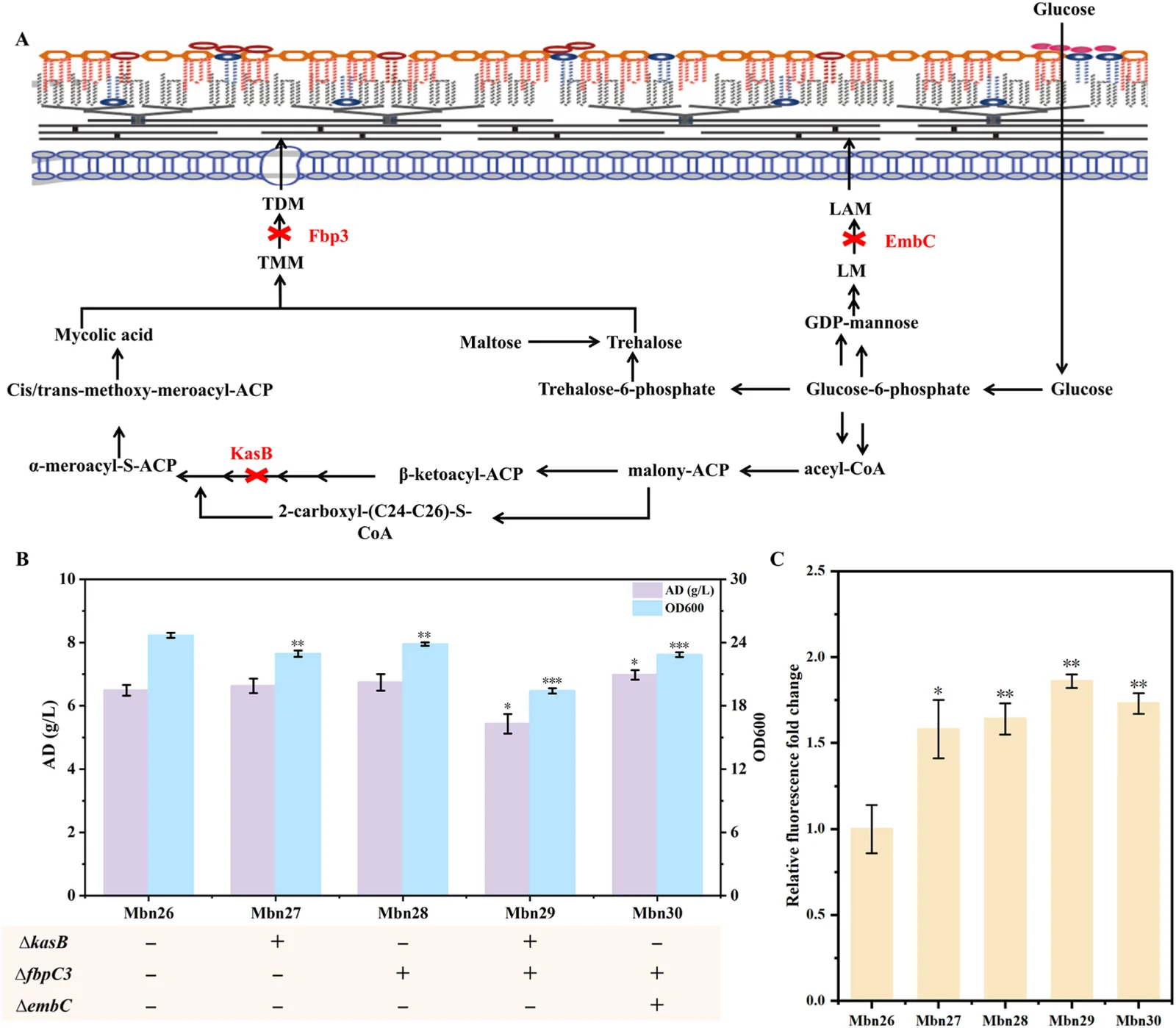
Cell envelope remodeling enhances cell permeability and AD production from phytosterols. (A) Schematic representation of the mycobacterial cell envelope biosynthesis pathways. (B) Effects of *kasB*, *fbpC3*, and *embC* deletions on AD production and cell growth. (C) Cell permeability analysis of engineered strains based on FDA uptake.

Previous studies have shown that moderate modulation of mycobacterial cell wall biosynthesis can improve sterol bioconversion efficiency in *M. neoaurum* [24,38]. The *kasB* encodes a β-ketoacyl-acyl carrier protein synthase involved in mycolic acid chain elongation and the regulation of cell wall mycolic acid composition, whereas *fbpC3* encodes an Ag85 complex-associated mycolyltransferase responsible for TMM-to-TDM conversion and mycolyl transfer to cell wall components [23]. Both genes are closely associated with mycolic acid biosynthesis and transport (Fig. 5A). Since mycolic acids are key structural components contributing to cell envelope hydrophobicity and integrity, *kasB* and *fbpC3* were first individually deleted from Mbn26, and a *kasB*/*fbpC3* double-deletion strain was further constructed. Compared with the *kasB* deletion strain Mbn27, the *fbpC3* deletion strain Mbn28 showed a higher AD titer of 6.74 g/L, while maintaining an OD_600_ of 23.66, indicating that *fbpC3* deletion promoted AD accumulation without markedly impairing cell growth. However, although the *kasB*/*fbpC3* double-deletion strain Mbn29 exhibited the highest FDA uptake, with a relative fluorescence fold change of 1.86, its AD titer decreased to 5.43 g/L and OD_600_ declined to 19.41 (Fig. 5B and C). The mismatch between the highest FDA signal and reduced AD production shows that permeability enhancement is beneficial only within an optimal range. Excessive disruption of mycolic acid chain elongation and mycolyl transfer likely weakened envelope integrity, causing growth defects and reducing whole-cell catalytic capacity.

To further enhance sterol uptake, the LAM assembly pathway was subsequently targeted. The *embC* is involved in LAM biosynthesis, and its deletion can weaken the cell envelope barrier and facilitate transmembrane transport [38]. The resulting strain Mbn30 showed a further increase in AD production to 6.98 g/L, while maintaining relatively stable cell growth (Fig. 5B). FDA uptake analysis showed that the relative fluorescence fold change of Mbn30 increased to 1.73 (Fig. 5C). Compared with *kasB*/*fbpC3* deletion, the *fbpC3*/*embC* combination improved permeability without causing a strong growth penalty, suggesting that simultaneous but moderate perturbation of mycolyl transfer and LAM assembly achieved a better balance between transport efficiency and cell robustness.

### 3.5 High-level production of androstenedione in a 5 L fermenter

**Fig. 6:**
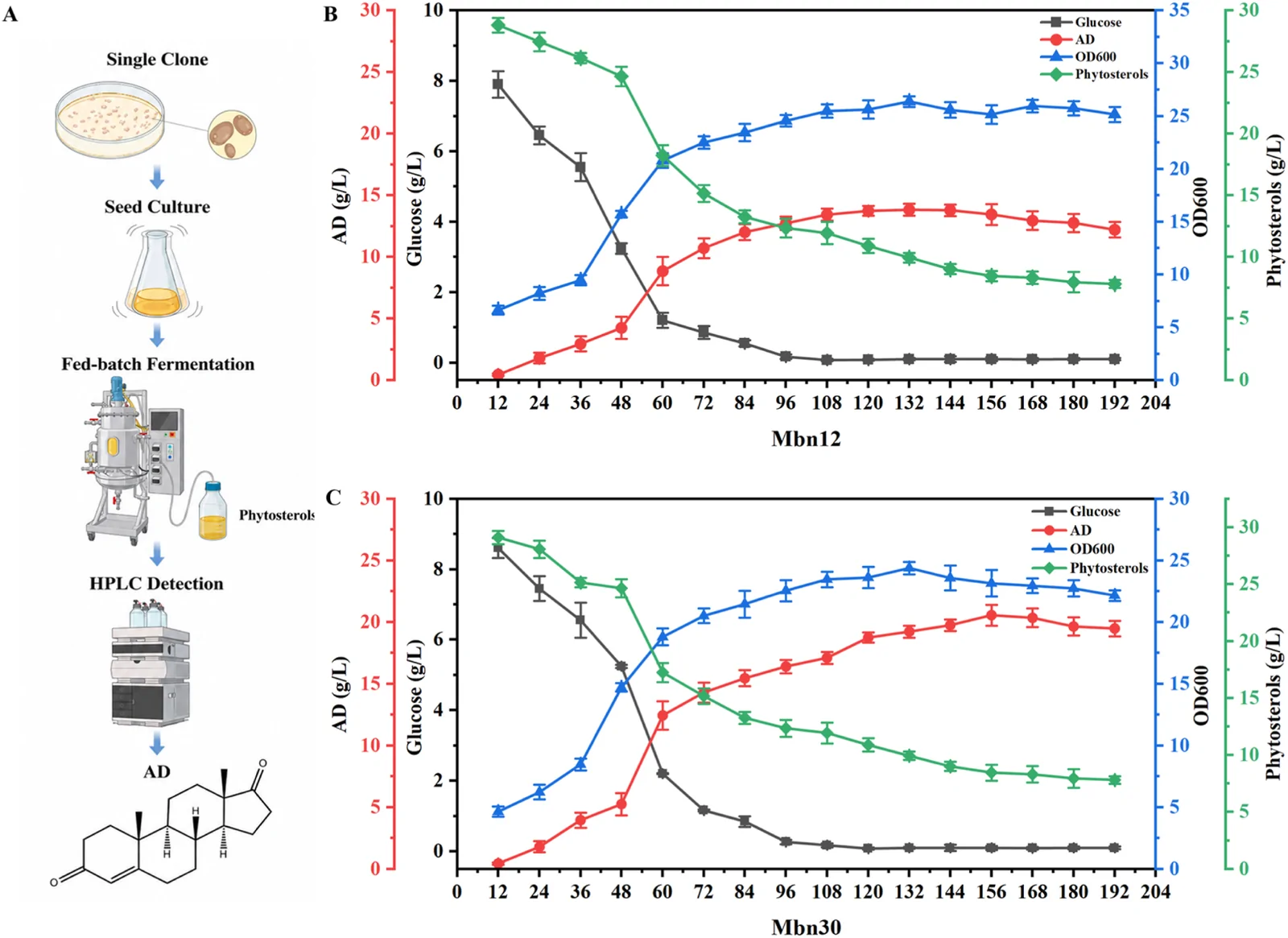
High-level production of AD from phytosterols in a 5 L fermenter. (A) Schematic workflow of fed-batch fermentation for AD production from phytosterols. (B) Time profiles of glucose consumption, AD production, cell growth, and residual phytosterols during fermenter-scale phytosterol biotransformation of Mbn12. (C) Time profiles of glucose consumption, AD production, cell growth, and residual phytosterols during fermenter-scale phytosterol biotransformation of Mbn30.

To evaluate scale-up applicability, Mbn12 and Mbn30 were subjected to phytosterol biotransformation in a 5 L fermenter using 30 g/L phytosterols as the substrate. Glucose consumption, cell growth, residual phytosterols, and AD accumulation were monitored under controlled conditions (Fig. 6A). Mbn12 grew normally in the fermenter, with OD600 gradually increasing and then remaining stable. Glucose was rapidly consumed during the early stage, accompanied by gradual phytosterol degradation and AD accumulation. The AD titer of Mbn12 reached 13.83 g/L, but further accumulation became limited at the late stage (Fig. 6B). This plateau indicates that blocking AD degradation alone was not sufficient under high-substrate conditions, where substrate transport, redox balance, and cellular catalytic capacity can become limiting.

In contrast, the final engineered strain Mbn30 showed markedly improved fermenter-scale performance. AD production increased rapidly after 48 h and reached 20.55 g/L at 156 h, representing a 48.6% improvement over Mbn12 (Fig. 6C). The corresponding molar conversion reached 98.1%, indicating efficient redirection of phytosterol-derived carbon flux toward AD. Compared with recent microbial AD-producing systems, Mbn30 exhibited a competitive production level under a moderate phytosterol loading. For example, Chang et al. produced 25.8 g/L AD from 50 g/L phytosterols using a two-step resting-cell bioprocess, while Li et al. achieved 38.3 g/L 4-AD from 80 g/L phytosterols in a 15 L fermenter. Although these studies reported higher absolute titers, they required substantially higher substrate loadings and showed lower molar conversions. In comparison, Mbn30 produced 20.55 g/L AD from 30 g/L phytosterols with a high molar conversion, demonstrating improved substrate-to-product efficiency (Table 1). Meanwhile, Mbn30 maintained a relatively stable OD_600_ during the later conversion stage, indicating that the additional engineering strategies did not compromise growth stability under scale-up conditions. The sustained consumption of phytosterols further suggested that transcriptional regulation, cofactor regeneration, and cell envelope remodeling cooperatively enhanced substrate utilization and whole-cell biotransformation. These results demonstrate that Mbn30 is a promising strain for efficient and scalable AD production from phytosterols.

**Table 1:** Recent advances in androstenedione production from phytosterols using microorganisms.

Host	Engineering strategy	Scale	Phytosterol loading	Titer	Time	Molar conversion	Ref.
*Mycobacterium* sp. B3805	High-concentration phytosterol microdispersion bioconversion	Shake flask	20 g/L	7.4 g/L	264 h	53.0%	[39]
*M. neoaurum*	Metabolic pathway regulation and two-step bioprocess	5 L fermenter	50 g/L	25.8 g/L	168 h	73.9%	[40]
*Mycobacterium* sp. HGMS2	Blocking endogenous *kstD* and *ksh* genes to reduce AD degradation	15 L fermenter	80 g/L	38.3 g/L	168 h	68.6%	[41]
*M. neoaurum*	Propionyl-CoA pathway enhancement	Shake flask	5 g/L	3.34 g/L	168 h	95.7%	[15]
*M. neoaurum*	Metabolic engineering and substrate pretreatment	Shake flask	10 g/L	9.29 g/L	72 h	98.3%	[14]
*M. neoaurum* LY-2	Multidimensional metabolic engineering	5 L fermenter	30 g/L	20.55 g/L	156 h	98.1%	This study

## 4 Conclusion

In this study, *M*. *neoaurum* LY-2 was systematically engineered to improve AD production from phytosterols for industrial application. Blocking competing C19 and C22 steroid branches established a selective AD-accumulating chassis, demonstrating that precise control of downstream steroid catabolism is essential for directing phytosterol-derived flux toward the desired intermediate. Further deletion of *fdmR* enhanced sterol side-chain degradation, while simultaneous reinforcement of the glyoxylate and methylcitrate cycles improved the assimilation of acetyl-CoA and propionyl-CoA generated during phytosterol catabolism. In addition, strengthening FAD supply, NAD^+^ regeneration, and H_2_O_2_ removal helped maintain redox balance during high-flux sterol oxidation. Moderate remodeling of the cell envelope further promoted phytosterol transport without markedly impairing cell growth. Through these combined modifications, the final strain Mbn30 produced 20.55 g/L AD from 30 g/L phytosterols in a 5 L fermenter, which was 48.6% higher than that of the basal AD-producing strain Mbn12. These results show that efficient AD production depends on the coordinated optimization of pathway flux, central metabolism, redox balance, and substrate transport. This work provides a practical engineering strategy for developing robust Mycobacterium-based cell factories for AD production and may also support the biosynthesis of other steroid intermediates from phytosterols.

## CRediT authorship contribution statement

**Liyang Xu:** Data curation, Investigation, Methodology, Visualization, Writing – original draft. **Chuxian Peng:** Investigation, Methodology. **Wenjing Pan:** Data curation, Investigation. **Yingjia Tong:** Formal analysis, Supervision, Validation. **Jinsong Shi:** Formal analysis, Supervision, Validation. **Zhenghong Xu:** Conceptualization, Project administration, Supervision. **Hui Li:** Conceptualization, Formal analysis, Funding acquisition, Methodology, Supervision, Writing – review & editing.

## Declaration of competing interest

The authors declare that they have no known competing financial interests or personal relationships that could have appeared to influence the work reported in this paper.
