# Supplementary material for: Combinatorial metabolic engineering of Mycobacterium neoaurum LY-2 for efficient androstenedione production from phytosterols
Source: Synth Syst Biotechnol. 2026 Aug 19;18:1–12. doi: 10.1016/j.synbio.2026.08.003 (PMC13520905; doi:10.1016/j.synbio.2026.08.003)
Supplement: Supplementary file 1 [file mmc1.docx]

**Supporting Information**

**Combinatorial Metabolic Engineering of *Mycobacterium neoaurum* LY-2 for Efficient Androstenedione Production from Phytosterols**

Liyang Xu^1^, Chuxian Peng^1^, Wenjing Pan^1^, Yingjia Tong^1^, Jinsong Shi^1^, Zhenghong Xu^2^, Hui Li^1,^ *

^1^ School of Life Sciences and Health Engineering, Jiangnan University, Wuxi 214122, China

^2^ College of Biomass Science and Engineering, Sichuan University, Chengdu 610065, China

* Corresponding author: Hui Li, E-mail addresses: lihui@jiangnan.edu.cn

Mailing address: School of Life Sciences and Health Engineering, Jiangnan University, 1800 Lihu Road, Wuxi, Jiangsu 214122, China.

**Contents**

**Supplementary Figures 1 to 3**

**Supplementary Tables 1 to 3**

**Fig. S1. Effects of FdmR and KstR deletion on cell growth and intracellular ATP levels.** (A) Growth profiles of Mbn12, Mbn12∆KstR, and Mbn12∆FdmR during phytosterol bioconversion. (B) Intracellular ATP contents of Mbn12, Mbn12∆KstR, and Mbn12∆FdmR at different transformation stages.

**Figure S2. Redox stress caused by enhanced phytosterol side-chain degradation.** (A) Extracellular H_2_O_2_ levels and (B) Relative intracellular ROS levels in Mbn12 and Mbn12∆FdmR-icl1-prpR during phytosterol conversion.

**Fig. S3. Oxidative stress responses of engineered strains during phytosterol bioconversion.** (A) Extracellular H₂O₂ levels. (B) Relative ROS levels

**Table S1. Description and sources of strains.**

| Strain | Description | Source |
| --- | --- | --- |
| ***Escherichia coli*** |  |  |
| *E.coli* JM109 | Cloning host, endA1 recA1 gyrA96 thi-1 hsdR17 (rₖ⁻ mₖ⁺) relA1 supE44 Δ(lac-proAB) | Invitrogen |
| ***Mycobacterium neoaurum*** |  |  |
| M. neoaurum LY-2 (Mbn0) | Wild-type or parental strain for phytosterol conversion | This study |
| Mbn1 | Mbn0 derivative with deletion of *kstD1* | This study |
| Mbn2 | Mbn0 derivative with deletion of *kstD2* | This study |
| Mbn3 | Mbn0 derivative with deletion of *kstD3* | This study |
| Mbn4 | Mbn0 derivative with deletion of *kstD4* | This study |
| Mbn5 | Mbn0 derivative with deletion of *kstD5* | This study |
| Mbn6 | Mbn0 derivative with deletion of *kstD1*-*kstD5* | This study |
| Mbn7 | Mbn6 derivative with deletion of *kshA1* | This study |
| Mbn8 | Mbn6 derivative with deletion of *kshA2* | This study |
| Mbn9 | Mbn6 derivative with deletion of *kshA1* and *kshA2* | This study |
| Mbn10 | Mbn9 derivative with deletion of *salA* | This study |
| Mbn11 | Mbn9 derivative with deletion of *opccR* | This study |
| Mbn12 | Mbn9 derivative with deletion of *salA* and *opccR* | This study |
| Mbn13 | Mbn12 derivative with deletion of *kstR* | This study |
| Mbn14 | Mbn12 derivative with deletion of *fdmR* | This study |
| Mbn15 | Mbn13 derivative overexpressing *icl1* | This study |
| Mbn16 | Mbn13 derivative overexpressing *prpR* | This study |
| Mbn17 | Mbn13 derivative co-overexpressing *icl1* and prpR | This study |
| Mbn18 | Mbn17 derivative overexpressing *ribA* | This study |
| Mbn19 | Mbn17 derivative overexpressing *ribB* | This study |
| Mbn20 | Mbn17 derivative overexpressing *ribC* | This study |
| Mbn21 | Mbn17 derivative overexpressing *ribD* | This study |
| Mbn22 | Mbn17 derivative overexpressing *ribF* | This study |
| Mbn23 | Mbn17 derivative overexpressing *ribH* | This study |
| Mbn24 | Mbn17 derivative co-overexpressing *ribB* and *ribC* | This study |
| Mbn25 | Mbn24 derivative overexpressing endogenous *ndhF* | This study |
| Mbn26 | Mbn25 derivative overexpressing endogenous *katA* | This study |
| Mbn27 | Mbn26 derivative with deletion of *kasB* | This study |
| Mbn28 | Mbn26 derivative with deletion of *fbpC3* | This study |
| Mbn29 | Mbn26 derivative with deletion of *kasB* and *fbpC3* | This study |
| Mbn30 | Mbn28 derivative with deletion of *embC* | This study |

**Table S2.** **Plasmids used in this study.**

| **Plasmids** | **Genotype or characteristic** | **Source** |
| --- | --- | --- |
| p2NIL | Shuttle vector of *E. coli* and *Mycobacterium* with blue-white screening marker *bla(M)*, Kan^R^ | Biofeng |
| p2NILB | p2NIL-derived plasmid containing the sucrose screening marker *SacB* | This study |
| p2NILB-KstD1UD | p2NILB-derived plasmid, carrying upstream and downstream homologous arms of *kstD1* | This study |
| p2NILB-KstD2UD | p2NILB-derived plasmid, carrying upstream and downstream homologous arms of *kstD2* | This study |
| p2NILB-KstD3UD | p2NILB-derived plasmid, carrying upstream and downstream homologous arms of *kstD3* | This study |
| p2NILB-KstD4UD | p2NILB-derived plasmid, carrying upstream and downstream homologous arms of *kstD4* | This study |
| p2NILB-KstD5UD | p2NILB-derived plasmid, carrying upstream and downstream homologous arms of *kstD5* | This study |
| p2NILB-KshA1UD | p2NILB-derived plasmid, carrying upstream and downstream homologous arms of *kshA1* | This study |
| p2NILB-KshA2UD | p2NILB-derived plasmid, carrying upstream and downstream homologous arms of *kshA2* | This study |
| p2NILB-SalAUD | p2NILB-derived plasmid, carrying upstream and downstream homologous arms of *salA* | This study |
| p2NILB-OpccRUD | p2NILB-derived plasmid, carrying upstream and downstream homologous arms of *opccR* | This study |
| p2NILB-KstRUD | p2NILB-derived plasmid, carrying upstream and downstream homologous arms of *kstR* | This study |
| p2NILB-FdmRUD | p2NILB-derived plasmid, carrying upstream and downstream homologous arms of *fdmR* | This study |
| p2NILB-KasBUD | p2NILB-derived plasmid, carrying upstream and downstream homologous arms of *kasB* | This study |
| p2NILB-FbpC3UD | p2NILB-derived plasmid, carrying upstream and downstream homologous arms of fbpC3 | This study |
| p2NILB-EmbCUD | p2NILB-derived plasmid, carrying upstream and downstream homologous arms of *embC* | This study |
| p2NILB-KshA1UD-Icl1 | p2NILB-derived integration plasmid for inserting the *icl1* expression cassette into the *kshA1* locus | This study |
| p2NILB-KshA2UD-PrpR | p2NILB-derived integration plasmid for inserting the *prpR* expression cassette into the *kshA2* locus | This study |
| p2NILB-FdmRUD-RibA | p2NILB-derived integration plasmid for inserting the *ribA* expression cassette into the *fdmR* locus | This study |
| p2NILB-FdmRUD-RibB | p2NILB-derived integration plasmid for inserting the *ribB* expression cassette into the *fdmR* locus | This study |
| p2NILB-KshA2UD-RibC | p2NILB-derived integration plasmid for inserting the *ribC* expression cassette into the *kshB* locus | This study |
| p2NILB-FdmRUD-RibD | p2NILB-derived integration plasmid for inserting the *ribD* expression cassette into the *fdmR* locus | This study |
| p2NILB-FdmRUD-RibF | p2NILB-derived integration plasmid for inserting the *ribF* expression cassette into the *fdmR* locus | This study |
| p2NILB-FdmRUD-RibH | p2NILB-derived integration plasmid for inserting the *ribH* expression cassette into the *fdmR* locus | This study |
| p2NILB-EmbCUD-NdhF | p2NILB-derived integration plasmid for inserting the *ndhF* expression cassette into the *embC* locus | This study |
| p2NILB-FbpC3UD-KatA | p2NILB-derived integration plasmid for inserting the *katA* expression cassette into the *fbpC3* locus | This study |

**Table S3. Primers used in this study.**

| **Primers** | **Sequences** | **Specifications** |
| --- | --- | --- |
| sacB-F | AACTCACGTTCACATATACCTGCCGTTCACTATTATTTAGTG | For p2NILB plasmid construction |
| sacB-R | CTGCGTCACTATTTGTTAACTGTTAATTGTCCTTGTTCAAGGATGC |  |
| Rp2NIL-sacB-F | AGTTAACAAATAGTGACGCAGATCCCGCAAGA |  |
| Rp2NIL-sacB-R | AGGTATATGTGAACGTGAGTTTTCGTTCCACTGAG |  |
| KstD1-U-F | CAGAATCCTGAGGCCGAGGAGATGATGTCGGC | For p2NILB-KstD1UD plasmid construction |
| KstD1-U-R | CCCCGATGTGGGGATACGGGTCTCCGGTCTG |  |
| KstD1-D-F | CGTATCCCCACATCGGGGAGTTCCTGCTTCGT |  |
| KstD1-D-R | TGACTTGACGGGCAGATCCGAGGGCAACTCGAG |  |
| Rp2NILB-KstD1-UD-F | GGATCTGCCCGTCAAGTCAGCGTAATGCTCTG |  |
| Rp2NILB-KstD1-UD-R | CCTCGGCCTCAGGATTCTGGCGTACCGTTCCTGTC |  |
| KstD2-U-F | CCGTTGTCGCGTGGCGGCCAACACATCATC | For p2NILB-KstD2UD plasmid construction |
| KstD2-U-R | TCGACATCGATGGGCATAGTAGAACACGTTATAGAATTGCAGTGCC |  |
| KstD2-D-F | CACTGCAATTCTATAACGTGTTCTACTATGCCCATCGATGTCGAGCAG |  |
| KstD2-D-R | AGCAAGGCCGGGCAATTCGACCGAGGTGGAC |  |
| Rp2NILB-KstD2-UD-F | CGAATTGCCCGGCCTTGCTGGTAATATCCAG |  |
| Rp2NILB-KstD2-UD-R | TGGCCGCCACGCGACAACGGTTAATTTGCGTG |  |
| KstD3-U-F | GTTGTCGCCATGGGAGGTCTCCAACCCCAGC | For p2NILB-KstD3UD plasmid construction |
| KstD3-U-R | CATCGATGGGCCAGGCCCACAACTGTGCCGG |  |
| KstD3-D-F | GTGGGCCTGGCCCATCGATGTCGAGCAGGC |  |
| KstD3-D-R | TTACCAGCAAGACAGTCTCCCACTCGGGTTTCGG |  |
| Rp2NILB-KstD3-UD-F | GGAGACTGTCTTGCTGGTAATATCCAGAACAATCCTGAATC |  |
| Rp2NILB-KstD3-UD-R | CCTCCCATGGCGACAACGGTTAATTTGCGTGATG |  |
| KstD4-U-F | CCGTTGTCGCGATGTGCCCAGCCCGATCGATCC | For p2NILB-KstD4UD plasmid construction |
| KstD4-U-R | CATTGTCTTCCAGCAGTCAACCCACTATAGCCACGTTTTG |  |
| KstD4-D-F | GTTGACTGCTGGAAGACAATGCCGGACCTGGCC |  |
| KstD4-D-R | GATATTACCAGCAACTGGTGAATGTGCGAGCGCCAC |  |
| Rp2NILB-KstD4-UD-F | CATTCACCAGTTGCTGGTAATATCCAGAACAATCCTGAATC |  |
| Rp2NILB-KstD4-UD-R | TGGGCACATCGCGACAACGGTTAATTTGCGTG |  |
| KstD5-U-F | CCGTTGTCGCCACGGGTGACCAACTCGCATTGCG | For p2NILB-KstD5UD plasmid construction |
| KstD5-U-R | GCCTGAGGTCCGCCTCCCCTTGCGTATCGAGT |  |
| KstD5-D-F | CAAGGGGAGGCGGACCTCAGGCGGCGATGAGGC |  |
| KstD5-D-R | TTACCAGCAAGCTCATCGACTACATCGCGCCGTC |  |
| Rp2NILB-KstD5-UD-F | GTCGATGAGCTTGCTGGTAATATCCAGAACAATCCTGAATC |  |
| Rp2NILB-KstD5-UD-R | GTCACCCGTGGCGACAACGGTTAATTTGCGTG |  |
| KshA1-U-F | CCGTTGTCGCCGAAGAGGCCAACGTCGGGC | For p2NILB-KshA1UD plasmid construction |
| KshA1-U-R | TGCGTGGTCAGGTGGGCCTCCCGTATCTGG |  |
| KshA1-D-F | GGAGGCCCACCTGACCACGCATGACGAGCGG |  |
| KshA1-D-R | GATATTACCAGCAAAGTTGATCGGCCTCAACGAG |  |
| Rp2NILB-KshA1-UD-F | GCCGATCAACTTTGCTGGTAATATCCAGAAC |  |
| Rp2NILB-KshA1-UD-R | GGCCTCTTCGGCGACAACGGTTAATTTGCGTG |  |
| KshA2-U-F | CCGTTGTCGCGACGTCCGCTATTTCGGAGCC | For p2NILB-KshA2UD plasmid construction |
| KshA2-U-R | GAACGTGCTAGGCAGATCAGGCGAACCGGCC |  |
| KshA2-D-F | GGCAGATCAGGCGAACCGGCCAACGGCCGTG |  |
| KshA2-D-R | GATATTACCAGCAATGTGGGCGAAGGCCCCGATG |  |
| Rp2NILB- KshA2-UD-F | CGCCCACATTGCTGGTAATATCCAGAACAATC |  |
| Rp2NILB- KshA2-UD-R | GCGGACGTCGCGACAACGGTTAATTTGCGTG |  |
| SalA-U-F | CAGTGAGGCGTTCGACGGCGAGTTCCTCGGG | For p2NILB-SalAUD plasmid construction |
| SalA-U-R | GTCATCAATCGCAAACCCAACGCCATCTCCCTC |  |
| SalA-D-F | GTTGGGTTTGCGATTGATGACCGAGTCGTCGGCCC |  |
| SalA-D-R | CTTGACGGGACCCAGAGTCTTTGCGCCGCATCG |  |
| Rp2NILB-SalA-UD-F | AAGACTCTGGGTCCCGTCAAGTCAGCGTAATGC |  |
| Rp2NILB-SalA-UD-R | GCCGTCGAACGCCTCACTGATTATAAAAACACTTCTCAGG |  |
| OpccR-U-F | GAGTCTGTCCCGACACGCAGACCATGCTCGACG | For p2NILB-OpccRUD plasmid construction |
| OpccR-U-R | CACCACGATAGGTGGCGGCGTACGTCAACAGTG |  |
| OpccR-D-F | TACGCCGCCACCTATCGTGGTGACGTGACAGGGCC |  |
| OpccR-D-R | GGCTTTGTTGCCAGTCGACCACGGTCATCGCC |  |
| Rp2NILB-OpccR-UD-F | GGTCGACTGGCAACAAAGCCGCCGTCCCGTC |  |
| Rp2NILB-OpccR-UD-R | CTGCGTGTCGGGACAGACTCTTTTGCTCGGTGGCC |  |
| KstR-U-F | CAGTGAGGCCGACGGTGACCTGCTGGCTACG | For p2NILB-KstRUD plasmid construction |
| KstR-U-R | ACACCTTAGGGTTGTGCTGGCGATGACATCTGCC |  |
| KstR-D-F | CCAGCACAACCCTAAGGTGTGAGGGGTGTTCGACACC |  |
| KstR-D-R | CTTGACGGGACCAGCCGATGTCCAAGGCCGAG |  |
| Rp2NILB-KstR-UD-F | ACATCGGCTGGTCCCGTCAAGTCAGCGTAATGCTCTGC |  |
| Rp2NILB-KstR-UD-R | GTCACCGTCGGCCTCACTGATTATAAAAACACTTCTCAGGATTCTGGC |  |
| FdmR-U-F | CAGTGAGGCGATATCGCCTCGCTGTCCACCCG | For p2NILB-FdmRUD plasmid construction |
| FdmR-U-R | CTTCAGACCGCTTCGTTCCTCCGGCCATCGGAAC |  |
| FdmR-D-F | GAGGAACGAAGCGGTCTGAAGGGCAGGCCGAC |  |
| FdmR-D-R | CTTGACGGGACCGAAGAACCGGGAGCACCGATGAAC |  |
| Rp2NILB-FdmR-UD-F | CGGTTCTTCGGTCCCGTCAAGTCAGCGTAATGCTCTGC |  |
| Rp2NILB-FdmR-UD-R | AGGCGATATCGCCTCACTGATTATAAAAACACTTCTCAGGATTCTGGC |  |
| KasB-U-F | CGTTGTCGCGTATGGCGGTGTCGGCGCTGG | For p2NILB-KasBUD plasmid construction |
| KasB-U-R | GTACTGCGATCTCCGAGCCAAACTTCTCGCGCAG |  |
| KasB-D-F | GTTTGGCTCGGAGATCGCAGTACATGAGGTGGGGAACG |  |
| KasB-D-R | AGCAAGGCCGGGCTTTGGCCTTGCGCTCCA |  |
| Rp2NILB-KasB-UD-F | GCCAAAGCCCGGCCTTGCTGGTAATATCCAG |  |
| Rp2NILB-KasB-UD-R | ACCGCCATACGCGACAACGGTTAATTTGCGTGA |  |
| FbpC3-U-F | CGTTGTCGCGGCCGCATCGTCGAGGTCAATTGGC | For p2NILB-FbpC3UD plasmid construction |
| FbpC3-U-R | CAGTCCGTCAAACGGCAACCAACCCACCTTTC |  |
| FbpC3-D-F | GGTTGCCGTTTGACGGACTGACCCGACACACC |  |
| FbpC3-D-R | GATATTACCAGCAAGCTGACGAACAGGACCGGGACTG |  |
| Rp2NILB-FbpC3-UD-F | GTTCGTCAGCTTGCTGGTAATATCCAGAACAATCCTGAATC |  |
| Rp2NILB-FbpC3-UD-R | CGATGCGGCCGCGACAACGGTTAATTTGCGTGATG |  |
| EmbC-U-F | ACCGTTGTCGCTCCGGCGCGCTGGCGCTGT | For p2NILB-EmbCUD plasmid construction |
| EmbC-U-R | GATCGACCCGCAACCGTCGGCCTGTGGTTTACGGATG |  |
| EmbC-D-F | GCCGACGGTTGCGGGTCGATCAGCCTCCATCTACCAG |  |
| EmbC-D-R | ATTACCAGCAATGTGGATCAGACCTACATCGCCGCGGCG |  |
| Rp2NILB-EmbC-UD-F | CTGATCCACATTGCTGGTAATATCCAGAACAATCCTGAATCGC |  |
| Rp2NILB-EmbC-UD-R | GCGCGCCGGAGCGACAACGGTTAATTTGCGTGATGG |  |
